# Safety Profile of Nebulized Ciprofloxacin‐Dexamethasone After Pediatric Airway Surgery

**DOI:** 10.1002/lary.70643

**Published:** 2026-05-27

**Authors:** Abbey L. Landini, Cyrus W. Abrahamson, Maya Frost, David Spielberg, Inbal Hazkani, Dana M. Thompson, Jonathan B. Ida, Sarah Maurrasse, Taher Valika

**Affiliations:** ^1^ Northwestern University Feinberg School of Medicine Chicago Illinois USA; ^2^ Department of Pediatrics‐Pulmonology Baylor University, Texas Children's Hospital Houston Texas USA; ^3^ Division of Pediatric Otolaryngology‐Head and Neck Surgery Ann and Robert H. Lurie Children's Hospital of Chicago Chicago Illinois USA; ^4^ Department of Otolaryngology‐Head and Neck Surgery Northwestern University Feinberg School of Medicine Chicago Illinois USA; ^5^ Department of Surgery, Division of Otolaryngology Yale University New Haven Connecticut USA

**Keywords:** adverse events, ciprofloxacin‐dexamethasone, nebulized therapy, pediatric airway surgery, safety, subglottic stenosis

## Abstract

**Objective:**

To evaluate the safety profile and patterns of use of nebulized ciprofloxacin–dexamethasone (CPD) in pediatric patients undergoing airway surgery.

**Methods:**

A retrospective chart review was performed of patients who underwent airway surgery at a tertiary pediatric center between 2019 and 2023 and received nebulized CPD 0.3%–0.1% postoperatively. Demographic, clinical, and procedural data were extracted. Exposure was defined as any postoperative nebulized administration. Adverse events were considered potentially CPD‐related if they occurred during administration or within 30 days of discontinuation without a better explanation. Outcomes included endocrine, metabolic, infectious, pulmonary, and inflammatory complications.

**Results:**

A total of 399 patients received nebulized CPD. Mean age was 46.2 months (range: newborn–20.7 years). Indications included subglottic stenosis (92.5%), post‐laryngotracheal reconstruction (5.3%), airway foreign body (2.0%), and supraglottoplasty (0.3%). Comorbidities were frequent, including neurologic conditions (47.6%), cardiac anomalies (41.6%), and bronchopulmonary dysplasia (25.1%). Most patients (93.4%) received CPD in the inpatient setting, for a mean duration of 11.7 days. No cases of adrenal suppression, glucose abnormalities, or bronchial hyperreactivity were identified. One patient had suspected airway fungal infection while receiving chemotherapy, though pathology was negative. Among the 85.7% of patients who underwent postoperative endoscopic evaluation, no significant inflammatory findings were observed.

**Conclusion:**

Nebulized CPD Was Uniformly Incorporated Into Postoperative Airway Management in This Large Pediatric Cohort and Well Tolerated, With no Significant Adverse Events Observed. These Findings Support the Safety of CPD in Medically Complex Children and Highlight the Need for Multicenter Prospective Studies to Evaluate Efficacy and Optimize Dosing Protocols.

**Level of Evidence:**

3

## 1 Introduction

Surgical management of pediatric airway stenosis plays a key role in restoring breathing, voice, and quality of life [1]. Whether performed endoscopically or through open reconstruction, postoperative healing is often complicated by inflammation, granulation tissue, and restenosis, which can lead to additional interventions and prolonged recovery [2, 3, 4]. Adjunctive therapies are frequently used to support airway healing and minimize complications, though evidence for many of these approaches remains limited [5, 6, 7, 8, 9, 10, 11].

Ciprofloxacin‐dexamethasone (CPD), a combination of topical fluoroquinolone and corticosteroid originally designed for otologic use, has become increasingly common in airway management protocols, despite being used off label per FDA guidelines [9]. Delivered via nebulizer or intratracheal drops, CPD is used with the intent of reducing inflammation and granulation tissue during the postoperative period [12, 13]. While a recent survey suggests widespread use among pediatric otolaryngologists, few studies have rigorously evaluated its safety and tolerability across diverse surgical settings, and no standardized dosing regimen currently exists [14].

Preliminary studies have reported potential benefits of CPD in pediatric airway management, including reduced granulation tissue and reduced time to tracheostomy decannulation [12, 13, 15, 16]. Adverse effects are infrequently reported and generally mild, with rare cases of systemic corticosteroid‐related effects such as adrenal suppression or Cushingoid features [14, 17]. A study in adults undergoing tracheal resection specifically evaluating CPD‐associated adverse effects found no attributable complications, further supporting the favorable safety profile suggested by pediatric studies [14, 18]. Moreover, recent work assessing socioeconomic factors and access to nebulized CPD in adult airway surgery suggests that, despite potential barriers, this therapy is largely accessible [19]. Nonetheless, the existing literature is limited by small sample sizes, procedure‐specific cohorts, and inconsistent dosing and delivery protocols. Importantly, no prior investigations have focused primarily on systematically evaluating safety or documenting adverse events in a pediatric population.

This study aims to characterize the real‐world use of nebulized CPD in a large, heterogenous cohort of pediatric patients undergoing airway surgery at a tertiary care center. We sought to evaluate its safety profile, describe patterns of administration, and identify any documented adverse effects. We hypothesize that adverse events will be rare, consistent with previous literature [14, 17, 18]. Findings from this work may support evidence‐based application of CPD in pediatric airway management postoperatively and inform future research on adjuvant therapies.

## 2 Materials and Methods

### 2.1 Study Design and Patient Selection

This study was a retrospective chart review of pediatric patients who underwent airway surgery at an academic tertiary care pediatric medical center between 2019 and 2023. This study was approved by the hospital Institutional Review Board.

Inclusion criteria included patients who underwent airway surgery and received nebulized CPD through a face mask as part of their postoperative care. Cases were identified via the electronic medical record (EMR). Patients were excluded if they did not receive CPD, if documentation regarding its administration was incomplete, or if CPD was administered via means other than a facemask.

### 2.2 Data Collection

Demographic variables collected included age, prematurity status, and comorbidities. Clinical and procedural data included the indication for surgery, location of airway pathology (e.g., supraglottic, glottic, subglottic), date of surgery, type of airway intervention, and context of CPD use (inpatient or outpatient). CPD use was identified by the documented administration of inhaled ciprofloxacin 0.3%‐dexamethasone 0.1%, typically formulated for otologic use. The administration frequency, duration, and clinical setting (inpatient or outpatient) of CPD was recorded.

Adverse events of interest included documented glucose abnormalities, signs of adrenal suppression, bronchial hyperreactivity, inflammatory findings, and fungal infections. These were determined by chart review of medical notes and laboratory results. Documentation of microlaryngoscopy and bronchoscopy (MLB) or flexible fiberoptic laryngoscopy (FFL) for postoperative evaluation was recorded.

### 2.3 Exposure and Outcome Definitions

Nebulized CPD exposure was defined as any documented postoperative administration of CPD via nebulizer, regardless of dose or frequency. The prescription for CPD nebulizers was uniform across all patients and involved nebulizing 1 mL of ciprofloxacin 0.3%‐dexamethasone 0.1% with 2 mL of normal saline, twice daily, for 14 days. Adverse events were classified as potentially CPD‐related if they occurred during CPD administration or within 30 days of discontinuation, provided no alternative cause was identified. Inflammatory outcomes (e.g., granulation tissue, edema) were abstracted from operative reports and postoperative endoscopic or clinical documentation in the medical record.

## 3 Results

A total of 399 pediatric patients received nebulized CPD following airway surgery during the study period. Patient demographics, surgical indications, and clinical characteristics are summarized in Table 1. The mean age was 46.2 months (3.9 years) and patients ranged in age from newborn to 20.7 years. Indications for CPD included subglottic stenosis in 369 patients (92.5%), post‐laryngotracheal reconstruction in 21 (5.3%), airway foreign body in 8 (2.0%), and postsupraglottoplasty in 1 (0.3%). Surgical pathology was most commonly located in the subglottis (97.7%), with fewer cases involving the bronchus (2.0%) or supraglottis (0.3%).

**TABLE 1:** Patient demographics, surgical indications, and clinical characteristics.

Characteristic	*n* (%) or Mean ± SD
Total patients	399
Age, months	46.2 ± 63
Prematurity	151 (37.8%)
Comorbidities	
Cardiac	166 (41.6%)
Craniofacial	47 (11.8%)
Syndromic	58 (14.5%)
Bronchopulmonary dysplasia (BPD)	100 (25.1%)
Neurological	190 (47.6%)
Oncologic	15 (3.8%)
Indication for CPD	
Subglottic stenosis	369 (92.5%)
Post‐laryngotracheal reconstruction	21 (5.3%)
Airway foreign body	8 (2.0%)
Postsupraglottoplasty	1 (0.25%)
Anatomic site	
Subglottis	390 (97.7%)
Bronchus	8 (2.0%)
Supraglottis	1 (0.25%)
Clinical setting	
Inpatient	373 (93.4%)
Outpatient	26 (6.5%)
Number of CPD administrations	3.3 ± 4.0
Duration of CPD use, days	11.7 ± 9.2
Postoperative airway evaluation	
Microlaryngoscopy and bronchoscopy (MLB)	244 (61.1%)
Flexible fiberoptic laryngoscopy (FFL)	98 (24.6%)
None	57 (14.29%)

Comorbid conditions were frequent in this cohort. Prematurity was present in 151 patients (37.8%). Neurologic conditions were the most common comorbidity, affecting 190 patients (47.6%), followed by cardiac anomalies in 166 (41.6%) and bronchopulmonary dysplasia in 100 (25.1%). Syndromic diagnoses were observed in 58 patients (14.5%), craniofacial anomalies in 47 (11.8%), and oncologic disease in 15 (3.8%).

The majority of patients (93.4%) received CPD in the inpatient setting, while smaller groups were treated following outpatient surgery (6.5%). CPD was administered a mean of 3.3 times (median 2, range 1–35), with an average duration of 11.7 days (median 10, range 2–114). Postoperative airway evaluation within 30 days of nebulized CPD administration was identified in the majority of patients. FFL was documented in 98 patients (24.6%), MLB in 244 (61.2%), and no postoperative endoscopic evaluation within 30 days in 57 (14.3%). Absence of a documented evaluation within this period did not necessarily indicate that no postoperative assessment occurred, but rather that it was not performed or recorded within the defined 30‐day search window.

Inflammatory changes were absent in all patients. No cases of bronchial hyperplasia, adrenal suppression, or glucose abnormalities were observed. One patient (0.3%) was documented as having a possible airway fungal infection, though this was not clearly attributable to CPD. This patient had negative pathology for a fungal infection and was concurrently receiving chemotherapy. There were no other adverse events reported (Table 2).

**TABLE 2:** Documented adverse events potentially related to nebulized CPD.

Adverse event	*n* (%)
Bronchial hyperplasia	0 (0.0%)
Adrenal suppression	0 (0.0%)
Glucose abnormalities	0 (0.0%)
Inflammatory changes	0 (0.0%)
Fungal infection	0 (0.0%)

## 4 Discussion

Nebulized CPD has emerged as an adjunct in the management of pediatric airway pathology, particularly following reconstructive surgery. By pairing the antimicrobial effects of ciprofloxacin with the anti‐inflammatory properties of dexamethasone, CPD is used anecdotally in many centers to mitigate granulation tissue formation and reduce the risk of restenosis [6, 14, 20]. Despite its growing use, published data on safety in children remain sparse, with most prior studies limited to small series, focused on adult patients, or not primarily analyzing CPD use. The primary aim of this study was to evaluate the safety profile of nebulized ciprofloxacin‐dexamethasone in a large pediatric patient cohort, as this represents an off‐label application with limited published safety data in the airway. Notably, there remains variability in clinical practice across the United States, with some institutions restricting its use for this indication due to the absence of robust safety data. Our study addresses this knowledge gap by reporting outcomes from the largest pediatric cohort to date treated with nebulized CPD after airway surgery.

Our findings build on prior work that has supported the safety of CPD. In adults, Abrahamson et al. demonstrated that outpatient nebulized CPD after endoscopic airway surgery was well tolerated and adhered to, with only transient, mild adverse effects reported by a small minority of patients [21]. Adult data after open airway reconstruction are similarly reassuring, with Chwa et al. observing no CPD‐attributable side effects in a postoperative inpatient regimen [18]. Pediatric reports also suggest tolerability, with Reddy et al. reporting no adverse events among 79 pediatric tracheostomy patients treated with CPD [13]. In contrast, a survey study by Shah et al. captured rare, but more serious side effects including Cushingoid features, mental status changes, hyperglycemia, and adrenal suppression, though the route of administration was not defined and events accrued across cohorts rather than within a defined cohort [14]. A case report also described transient hyperglycemia in a child with Type 1 diabetes following off‐label intranasal ciprofloxacin–dexamethasone administration, with blood glucose levels increasing by approximately 40–50 mg/dL during treatment before normalizing after discontinuation [17]. Importantly, none of the patients in our cohort had diabetes, which may limit our ability to detect similar metabolic effects in susceptible populations. By focusing specifically on nebulized CPD in a large pediatric population with standardized dosing, our study extends this literature and provides pediatric‐specific safety data.

In this retrospective cohort of 399 patients, nebulized CPD was administered uniformly following airway procedures, most commonly for subglottic stenosis and post‐laryngotracheal reconstruction. CPD was typically prescribed in the inpatient setting and continued for an average of 12 days, consistent with the treating surgeon's routine postoperative practice. Despite the broad use of CPD across a heterogeneous population, adverse effects were exceedingly rare. No patients developed adrenal suppression, glucose abnormalities, or bronchial hyperreactivity. Only one case of a suspected fungal infection was documented in a patient who was concurrently receiving chemotherapy and found to have negative pathological findings. These results are consistent with the existing literature reported by Abrahamson et al., Chwa et al., and Reddy et al., collectively supporting a favorable safety profile [13, 18, 21].

The high prevalence of comorbid conditions in this cohort further strengthens the safety observations. Nearly half the patients carried neurological diagnoses, more than 40% had cardiac anomalies, and one quarter had bronchopulmonary dysplasia. Syndromic and craniofacial abnormalities were also well represented. Given the vulnerability of these subgroups to systemic corticosteroid effects, the absence of clinically significant complications provides reassurance regarding the tolerability of CPD in medically complex children.

Our findings also provide insight into patterns of practice. Most patients underwent at least one postoperative endoscopic evaluation, either with FFL or MLB, allowing for direct assessment of airway healing. Notably, no patient demonstrated significant inflammatory findings postoperatively. This observation is in line with the theoretical rationale for CPD use in reducing mucosal edema and granulation, though causality cannot be established in the absence of a comparison group. The uniform prescription of 1 mL of ciprofloxacin 0.3%‐dexamethasone 0.1% nebulized in 2 mL of normal saline twice daily further strengthens the generalizability of the safety profile observed and is consistent with previous studies, though future studies may refine optimal dosing and duration of use.

This study is limited by its retrospective design, reliance on documentation for adverse event detection, and absence of a comparator group not treated with CPD. Subclinical endocrine or metabolic effects may not have been captured in the medical record. In addition, outcomes related to efficacy, such as rates of restenosis or time to decannulation, were not within the scope of this analysis. As this was a single‐institution study, the generalizability of our findings may be limited. Future research should focus on prospective, multicenter studies that can evaluate both the safety and efficacy of CPD with standardized protocols.

## 5 Conclusion

In this large, single‐institution cohort, nebulized CPD was well tolerated following airway surgery, with no clinically significant adverse events identified. Safety was maintained even among children with substantial comorbidities, including cardiac, neurological, and pulmonary conditions. These findings support the continued use of CPD as an adjunct in postoperative airway management and underscore the need for prospective studies to establish standardized dosing protocols and evaluate efficacy outcomes such as restenosis and decannulation.

## Funding

The authors have nothing to report.

## Conflicts of Interest

The authors declare no conflicts of interest.

## Data Availability

The data that support the findings of this study are available on request from the corresponding author. The data are not publicly available due to privacy or ethical restrictions.
